# Prevalence of Chronic Pain After Spinal Surgery: A Systematic Review and Meta-Analysis

**DOI:** 10.7759/cureus.41841

**Published:** 2023-07-13

**Authors:** Hotoon S Alshammari, Abdullah S Alshammari, Sulaiman A Alshammari, Shaik Shaffi Ahamed

**Affiliations:** 1 College of Medicine, King Saud University, Riyadh, SAU; 2 College of Medicine, AlMaarefa University, Riyadh, SAU; 3 Department of Psychiatry, Eradah Hospital, Ministry of Health, Riyadh, SAU; 4 Department of Family and Community Medicine, College of Medicine, King Saud University, Riyadh, SAU; 5 Department of Family and Community Medicine (Biostatistics), College of Medicine, King Saud University, Riyadh, SAU

**Keywords:** post-surgical spine pain, spinal surgery, laminectomy, discectomy, lumbar disc herniation

## Abstract

Degenerative disc disease and low back pain are common challenges that persist even after a discectomy. However, characterizations and quantifications of these illnesses from the patients' perspective are insufficient. We aimed to perform a systematic review of the literature and meta-analysis to determine the frequency of chronic pain after spinal surgery. We searched MEDLINE (PubMed), Google Scholar, and the Saudi Digital Library to retrieve research articles describing the frequency of persistent back pain, reoccurring disc herniation, and undergoing another operation following primary lumbar discectomy. We excluded articles that did not disclose the proportion of patients who experienced ongoing back or leg pain for over six months after the operation. We included 16 studies evaluating 85,643 patients. The pooled prevalence of persistent pain was 14.97% (95% confidence interval: 12.38-17.76). With all advancements in technology and operation techniques, many patients (14.97%) still have failed back surgery syndrome. Appropriate preoperative communication and multidisciplinary and coordinated treatment strategies yielded the best results.

## Introduction and background

Low back pain (LBP) is a frequent symptom globally; an estimated 60-80% of people will have LBP at some point in their life [1]. LBP symptoms persist for over three months in 10% of patients [2]. The high prevalence of LBP among patients has led to a rise in the number of surgical procedures performed in recent decades. Over one million spine operations, including 210,407 lumbar fusion surgeries, were conducted in the USA in 2002 [3,4]. In 2004, spinal fusion surgery cost over $16 billion [5]. The lumbar spinal operation has a 10% to 46% failure risk [6]. Despite improvements in surgical methods and technology, there will likely be an increasing number of patients who have failed back surgery syndrome (FBSS) [7]. Many terms have been coined to describe these conditions. In the 1970s, "post-laminectomy syndrome" and "failed back surgery syndrome" were coined to discuss ways to describe persistent pain after surgery. Nevertheless, these ambiguous phrases encompass various meanings and refer to disorders involving chronic pain before surgery that recur or persist. FBSS has been recognized since the commencement of spinal surgery. During the surgical end stage, after one or more operations on the lumbar neuroaxis, gives pain relief without any effect as described by Follett and Dirks [8]. The term "FBSS" refers to spine surgery that falls short of its intended outcome due to inadequate patient selection [9]. "Post-surgical spine syndrome" is a chronic or novel type of pain that occurs after spinal surgery. It may be related to nerve root injury, compression, arachnoiditis, epidural fibrosis, adjacent-level degeneration, and spinal instability. Complex regional pain syndrome is a chronic and sometimes progressive condition that occurs after spinal surgery. However, the precise cause and frequency of this rare condition remain unknown [10-12]. The proposed replacement terminology for FBSS is "persistent spinal pain syndrome" (PSPS). The International Association for the Study of Pain (IASP) task force suggested coding strategies and subtypes to improve PSPS. In 2019, the IASP recommended replacing FBSS with "chronic pain after spinal surgery," which will be included in the International Classification of Diseases (ICD)-11 [13]. The stigma and being marginalized have had devastating effects on these patients, including the deterioration of the trust of patients experiencing pain in healthcare professionals [14], quality of pain care [15], perceived injustice [16], and a worsening of stress [17] such as depression [18]. Medical opioid users face social stigma, including derogatory language. Individuals who use opioids for medical purposes may be ashamed owing to the misguided attempt to curb the problem of illicit opioids [19,20]. An accurate assessment is required to manage the symptoms of this difficult group of patients because of the potential widespread of this condition. In some countries, insurance companies rely on the diagnosis to determine treatment eligibility. For example, many insurers require FBSS or the synonymous ICD-10 term "post-laminectomy syndrome" diagnosis before paying for spinal cord stimulation treatment. The use of the new ICD-11 nomenclature, which has certain advantages over the more broad IASP terms, may enhance communication of patients' medical circumstances, which may be likely to respond to interventional pain and neurological stimulation therapies. Therefore, we aimed to conduct a systematic literature review and meta-analysis to determine the prevalence of chronic pain after spinal surgery.

## Review

Search strategy

We identified search terms and combined them with appropriate Boolean operators. We searched MEDLINE (PubMed), Google Scholar, and the Saudi digital library for research articles published in English. The search sequence submitted was as follows: “Failed back surgery syndrome,” OR “Pain recurrence after discectomy,” OR “Post-surgical spine syndrome,” OR “Repeat spinal surgery,” OR “Disc herniation requiring reoperation,” OR “Persistent spinal pain syndrome,” OR “Post-laminectomy reherniation relapse pain,” OR “Chronic pain after spinal surgery,” OR “Chronic pain after back surgery,” OR “Post spinal surgery syndrome,” OR “Post-laminectomy syndrome.” The search was conducted in April 2023 and was limited to publications between 2010 and 2022. We followed the Preferred Reporting Items for Systematic Reviews and Meta-Analyses (PRISMA) of observational studies [21,22]. We screened the references from the included studies and identified additional primary studies not previously identified. We imported the retrieved citations into EndNote 20 software (Clarivate, Philadelphia, PA, USA) and removed duplicates.

Inclusion and exclusion criteria

We included research articles written in English that (1) were published in peer-reviewed journals; (2) investigated the prevalence of pain that persisted for more than six months after spinal surgery; (3) had a reported cross-sectional, cohort, or longitudinal design; (4) had a defined sample; and (5) evaluated at least one of the primary clinically significant outcome variables (such as pain or functional status) with a valid instrument; (6) their patients were individuals with sciatica due to a herniated disc; and (7) scored at least 5 out of 9 on the Joanna Briggs (JBI) checklist [23]. We excluded case reports, case series, technical notes, conference abstracts, qualitative or review articles or commentaries, letters to the editor, studies with insufficient data, laboratory studies, and experimental studies.

Data collection process

Two reviewers (HS and AS) independently assessed the electronic search titles and abstracts and obtained complete papers. If no consensus could be achieved, a third reviewer (SA) was consulted to determine whether the article should be included or excluded. The entire texts of all articles preserved from the first stage were reviewed for inclusion and exclusion criteria in the second stage.

Quality assessment

Since the "prevalence" estimate is the most important one, the JBI Critical Appraisal Checklist was chosen to assess validity compared with other critical appraisal tools for systematic reviews of epidemiologic studies [24,25]. It assigns one score for each "yes" answer to nine questions on the quality of the study (yes, no, unclear, not applicable). These were added up for each article to provide a final JBI score. We used a cutoff value of 5/9 for every study to choose which studies to include. We independently assessed the quality of each study, and discrepancies were resolved through discussion and adjudication.

Data synthesis

We compiled tables of the retrieved studies and their key results. The extracted data consisted of authors, publication year, research, population, design (prospective versus retrospective), follow-up period, sample size (male/female), age, type of surgery, the measure of interest prevalence and tools to identify it, satisfaction, risk factors, the prevalence of persistent back or leg pain, and quality assessment score. If the prevalence was analyzed by subgroups such as sex, we included this column in the table. The project protocol was registered in PROSPERO (CRD42023418143).

Statistical analysis

We conducted a meta-analysis using MedCalc for Windows version 15.0 (MedCalc Software, Ostend, Belgium) to estimate the combined (pooled) prevalence of persistent pain after spinal surgery. Forest plots were used to graphically show the pooled prevalence (using fixed- and random-effect models) of the studies included in the meta-analysis. Heterogeneity in the pooled data was assessed using Cochran’s Q and I2, indicating the total variation percentage across the studies. A cut-off value of I2 > 50% was used to rule out higher levels of unexplained variability in the effect sizes. The significance of publication bias was assessed using Egger’s test. The precision of estimates was reported using 95% confidence intervals.

Results

**Figure 1:**
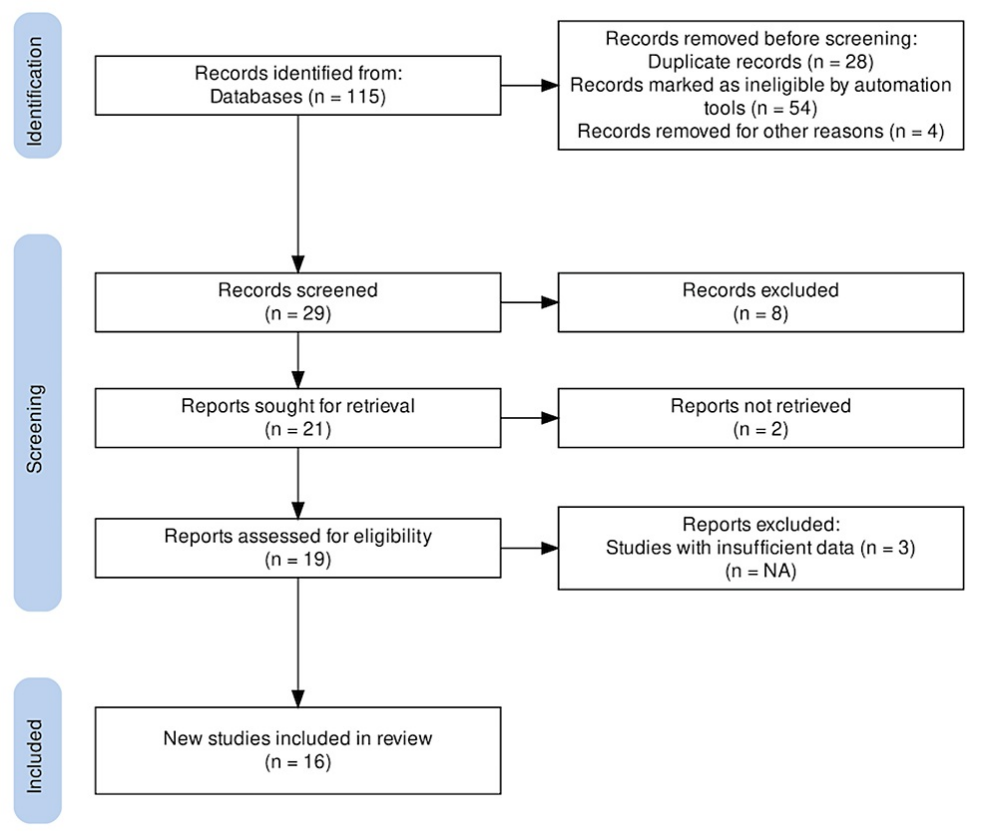
PRISMA flow diagram of included studies PRISMA: Preferred Reporting Items for Systematic Reviews and Meta-Analyses

The initial search yielded 115 published studies. We excluded 86 duplicate articles. The remaining 29 publications had their titles and abstracts reviewed; eight were disqualified after the screening. Twenty-one reports were retrieved; however, two articles were not retrieved. Three of the 19 studies assessed for eligibility were excluded for various reasons. Articles were also disqualified if they failed to provide the proportion of patients who experienced ongoing back or leg pain after spinal surgery were also excluded. Finally, 16 studies were included [26-40]. The studies considered are depicted in a PRISMA flowchart, which is shown in Figure 1.

Table 1 presents an informative summary of the studies and their patients.

**Table 1:** The characteristics of included studies and patients of spinal surgery

Author’s surname	Year of publication	Study population	Country	Study design (Prospective/ retrospective)	Follow-up period	Sample size M/F 84951 Patients	Age (years)	Type of surgery	Measure of interest	Satisfaction	Risk factors	Prevalence (long-term persistent back or leg pain). Other outcomes, recurrence	QA (JBI)
														Bugis SM et al. [9]	2022	824 discectomy/laminectomy King Abdulaziz University Hospital, Jeddah	Saudi Arabia	Retrospective	10 years	231 (Female=52.4%, Male=47.6%)	18-80 (50-70 most affected)	discectomy or laminectomy	Oswestry Low Back Pain Disability Questionnaire (ODI)	70%	87% BMI >30 , 20%diabetic, Smoker)	Severe pain (21.6%), assistance with personal care (13.9%) (1 to more than 3 surgeries)	9
																												Majed Ali et al. [40]	2021	spine surgery at Al-Kuwait University Hospital, Sana’a. October 1, 2018, until October 31, 2019	Yemen	Retrospective	One year	283 (female to male ratio, 1.0: 1.6)	31-60 Mean age 51.1	Lamin/dissect (64.5%), laminectomy (16.1%), fusion (13%), foraminotomy 3.2%	North et al. 1991, definition. (1)	NA	NA	11%, female/male ratio 1.0:1.6 most common age 40-49 years (mean 50.3)	7
Schoell K et al. [26]	2019	The nationwide insurance carrier Humana patients underwent lumbar spine surgery 2007-2015	USA	Retrospective	One year	70,581, records. Male= 48.5%, female=51.5%	Average age 64.4.	Laminectomy, discectomy, fusion.	Records of dural tear, damage to nervous tissue, cauda equina syndrome, neurogenic bowel/bladder, and FBSS, depression	NA	Depression 20.9%	15.05%, highest in multilevel procedures and posterior fusion. (F = 15.8%; M = 14.3%). highest at age 45-64 years and over 80 Depression was risk for FBSS.	7																												
														Lee YC et al. [29]	2019	Patients underwent spinal fusion in past 5 years	UK	Retrospective	Minimum 12 months (mean 42) (range, 12-77).	317 Male=45%, Female= 55%	56.7 ± 12.2	lumbar fusion surgery	FABER test, Thigh thrust, Iliac distraction test, Gaenslen’s test, and pain relief of >70% SIJ block	NA	NA	12.0% (average time to onset 22 months)	6
																												Weir S et al. [30]	2017	Population-based cohort linked to Clinical Practice Research Datalink (CPRD) and Hospital Episode Statistics (HES) underwent lumbar surgery 1997- 2012	England	Retrospective	Two years	10216 Male=49.1%	aged 18 plus	Lumbar surgery	patients records of further interventions, surgery/specialist pain clinics	NA	NA	20.8% increasing Healthcare usage/cost significantly	7
																																										Inoue S et al. [36]	2017	Internet survey of a nationally representative sample by Macromill Inc., a leading company in Japan having over 2,600,000 clients, December 2012	Japan	Retrospective	10 years	1842 Males= 321, Females=521	Mean age 42.6 (range 20-89)	lumbar surgery	EuroQol-5 (2) Kessler psychological distress scale (K6) (3)	78.40%	multiple surgeries, Spinal fusion	20.60%	7
																																																								Matsumoto M et al. [35]	2013	Patient underwent Microendoscopic discectomy (MED)	Japan	Retrospective	Mean 3.6 years range (2.0–6.5)	344 (males=213, Females=131)	Mean age 39.3 (range, 11-82)	Microendoscopic discectomy MED	Japanese Orthopedic Association (JOA) Score	NA	NA	10.80%	7
Desai A et al. [27]	2012	Cohort of first-time open discectomyTrial (SPORT) at 13 medical centers with multidisciplinary spine practices in 11 states. March 2000 to November 2004	USA	Retrospective	Mean 41.3 ± 14.6 months.	792 Female=43%	Mean age 40.7 ± 10.8	Standard first-time open discectomy	ODI SF-36 SCI Sciatica bothersomeness index	NA	NA	Rate of reoperation 2.7% Significant differences in age and race, and disability and treatment preferences. No differences in sex, BMI, smoking, diabetes or hypertension), or herniation level or type. No differences among centers in outcome at 4 years.	7																																																								
Ahsan K et al. [38]	2012	Records of prolapse of lumbar intervertebral discs PLID, and revision discectomy, Sheikh Mujib Medical University, Dhaka	Bangladesh	Retrospective	one to four years	416 PLID n=398, men73%, women=27% Recurrent PLID n=18 (men 78%, women 22%)	primary PLID aged 19 to 60 (mean, 39) Recurrent PLID aged 28 to 50 (mean, 40)	Primary and revision discectomy	Visual analogue score (VAS) Macnab criteria. ODI	NA	NA	15% no significant difference between revision and primary discectomy	7																																																								
														Marquardt G et al. [31]	2012	Reports of Far-lateral extraforaminal lumbar disc herniation (FELDH) surgery; 1989-2008 at Neurosurgical Clinic, Johann Wolfgang Goethe-University, Frankfurt	Germany	Retrospective	mean 146 months	138 patients (men 54.3%, women 45.7%)	Average age 56 (range 22-80).	Far-lateral extraforaminal lumbar disc herniation (FELDH) surgery	Standardized telephone interviews/structured questionnaires. ODI, MacNab criteria	75.9% excellent, 18.4% good	NA	27.6%. mainly sensory disturbance.	7																												
Lubbers T et al. [32]	2012	patients, with foraminal and extraforaminal lumbar disc herniation at L5-S1 treated by Percutaneous endoscopic lumbar discectomy (PELD), Sept 2004-April 2010.	Germany	Retrospective	mean 3.6 ± 2.0 (range 1-7 years)	22 patients: (males=60%, females=40%)	The mean 53.8 ± 10.5 (range 31-70)	Percutaneous endoscopic lumbar discectomy (PELD)	VAS, ODI, Macnab criteria	81.80%	NA	9%	5														
Wang M et al. [34]	2012	patients treated with MED, Jan 1999-Dec 2000. Department of Orthopaedics, Xinqiao Hospital, Third Military Medical University, Chong Qing	China	Retrospective	10-year	151 (87 males, 64 females	average age 39 (ranging 15 to 71)	microendoscopic discectomy (MED)	MacNab criteria The Mochida’s method	79% excellent, 12.9% good, 67% maintain occupations	NA	5%	7														
Chumnanvej S et al. [39]	2011	Full endoscopic discectomy patients, Jul 2008-Jan 2010 Ramathibodi Hosp, Faculty of Medicine, Mahidol University, Bangkok	Thailand	Prospective	Mean 23 months	60 patients (male=27, female=33)	Mean 46.3 ± 11.3 (ranged 18 to 70)	Full-endoscopic lumbar discectomy via Interlaminar approach	VAS,ODI, Macnab criteria	91.60%	NA	8%	6
														Parker SL et al. [28]	2010	patients' data of primary, single-level lumbar hemilaminotomy/discectomy	USA	Retrospective	Mean 37.3 months	111 patients M/F	Age	Discectomy	Institutional billing/accounting records for diagnoses and costs.	NA	NA	32%	5
																												Silverplats K et al. [33]	2010	Lumbar disc herniation surgery patients, September 1996 and March 2002	Sweden	Prospective	2 and 5-10 (mean 7.3) years	171 patients (women=44% male=56)	mean age 39 ± 11	Lumbar disc herniation surgery	VAS, ODI, Zung Depression Scale	72% satisfied 24% partly satisfied	NA	23%	8
Bakhsh A [37]	2010	Records of lumbar disc surgery Fauji Foundation Hosp, Rawalpindi, 1995-2004.	Pakistan	Retrospective	10 years	68 (women=44, men=24)	4th decade of life 30.8%, 5th 33.3%	lumbar disc surgery	Interview and physical exam.	NA	NA	15%	5																												

**Figure 2:**
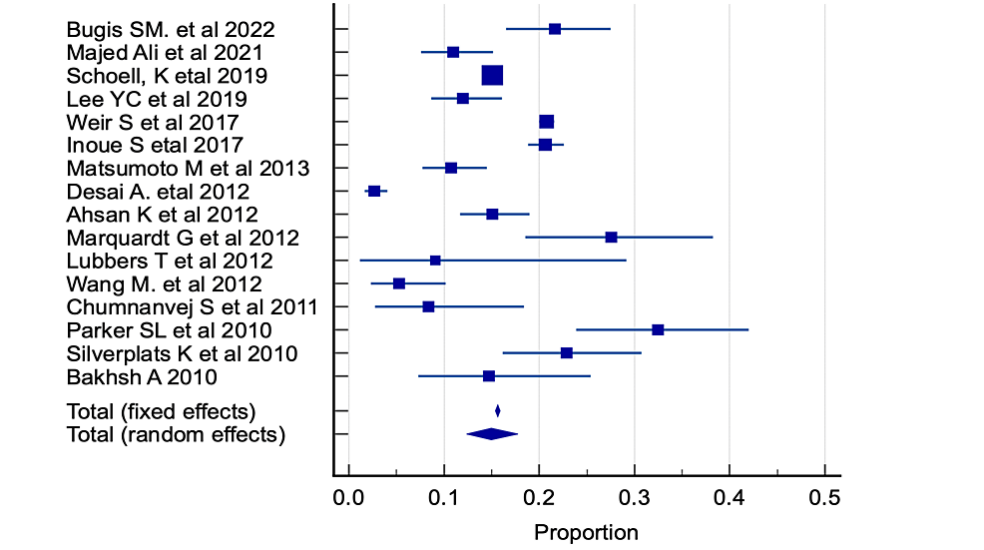
Forest plot for studies showing the prevalence of persistent pain in each study and pooled prevalence

The 16 articles included spanned approximately a decade from 2010 to 2022. The study population consisted of 70,581 (range 22-70,581) patients from various countries. The studies covered experiences of centers in the USA [26-28], United Kingdom [29,30], Germany [31,32], Sweden [33], China [34], Japan [35,36], Pakistan [37], Bangladesh [38], Thailand [39], Saudi Arabia [9], and Yemen [40]. Two studies were prospective, and the follow-up duration was between one and ten years. Some studies surveyed large populations, such as the 85,643 Humana insurance holders who had surgery on their lumbar spine between 2007 and 2015. Additionally, the linked Clinical Practice Research Datalink and Hospital Episode Statistics (CPRD-HES) investigated a population-based cohort of 10,216 patients who had lumbar operations in England between 1997 and 2012. In 2012, a nationally representative sample of 1842 Japanese people was surveyed online. The male-to-female ratios varied, with half of the studies having more male patients in their samples and the other half having the opposite. The patients’ age in the studies varied widely, with most studies covering adults aged 18-80, with a mean age of 39.0-64.4. Matsumoto et al. [35] and Wang et al. [34] studies included young patients aged 11-15. Two studies included children, adolescents, and adults [35,34]. There was considerable methodological variability, and the data extracted from different studies were heterogeneous. The following studies used the Oswestry LBP Disability Questionnaire (ODI) [9,27,38,31,32,39,33]. The visual analog scale (VAS) was used in [38,32,39,33]. The Japanese Orthopedic Association Score for LBP (JOA score) was used in [35]. The sciatica bothersomeness index was used to assess recurrent pain and disability [27]. The EuroQol-5 dimension (EQ-5D) and the Kessler Psychological Distress Scale (K6) were used to measure health-related quality of life (QOL) [41], the Short Form 36 questionnaire (SF-36), and Zung Depression Scale to assess depression. Three studies based their outcomes on physical examinations and interviews [37]. Estimates of persistent postoperative pain were derived from patients' medical records that detailed their presentation for additional interventions, surgery, or attendance at specialized pain clinics and from institutional billing and accounting records used to determine the cost of these measures [28,30]. Patients’ satisfaction with pain control after spinal surgery was high, ranging from 70 to 91% [9,36,31,32,34,39,33]. However, 5-27.6% of patients with spine surgery experienced postoperative pain despite advances in surgical techniques. Long-term outcomes of laminectomy and discectomy were measured using the ODI, VAS, and Macnab criteria. Silverplats et al. (2010), in a prospective study of 171 patients, discovered that 23% experience long-lasting problems [33]. The duration of sick leave was a clinically significant predictor of the primary outcomes, with the ability to change the odds for a satisfactory objective and subjective outcome from approximately 50% (three months leave) to 80% (two months leave). Additionally, the length of sick leave was a significant predictor of secondary outcomes, such as the ability to work and the necessity of medication for pain relief. Bugis et al. in 2022 [9] reported in a retrospective study of 231 patients that 21.6% complained of severe pain and 13.9% required assistance with personal care. FBSS was most common in patients aged 50-70, with a slight female predominance (52.4%). Most patients (87%) were overweight, and 20% were diabetic. Many patients (82.7%) could lead normal sexual lives, and 68.4% reported no sleep disturbances. Neurosurgeons performed surgeries in 95.2% of patients and orthopedic surgeons in the remaining 4.8%. Patients had anywhere from one back surgery to more than three. Various therapeutic modalities have been used to control LBP in patients with FBSS. Sixty percent of patients utilized physiotherapy, whereas 50.2% were prescribed medication. A total of 75.8% of all prescriptions were for nonsteroidal anti-inflammatory medicines (NSAIDs). The principal operating surgeon managed postoperative pain in 40% of cases. Adequate pain treatment was related to physiotherapy, pain service consultation, and medications. In a retrospective telephone interview research by Marquardt et al. (2012) involving 87 patients who had minimally invasive surgery (lateral approach), it was discovered that 27.6% of patients experienced persistent residual symptoms, with sensory disturbances being the most prevalent concern [31]. They concluded that the lateral approach is safe with low complication rates. Ahsan et al. (2012) [38] reviewed the primary and revision discectomy over 1-4 years. They discovered that 15% of patients who underwent primary discectomy had less than satisfactory outcomes, compared with 23% of those who underwent revisions. Clinical outcomes were unaffected by age, sex, smoking status, occupation, herniation degree, or pain-free interval. Complications included foot drop and dural tear. Lubbers et al. (2012) [32] studied 22 patients who underwent percutaneous endoscopic discectomy and discovered that 9% of patients required open surgery due to recurrent disc herniations. They concluded that the approach is a successful therapeutic option for L5-S1 foraminal and extraforaminal disc herniations in carefully allocated patients. Chumnanvej et al. [39] discovered that 8% of 60 patients had postoperative pain after a full-endoscopic lumbar discectomy. They concluded that the procedure was safe and effective; however, the learning curve is steep. Proper surgical training and careful patient selection in early cases are crucial for a successful recovery. Patients can expect less postoperative pain and a short absence from work. Desai et al. (2012) [27] studied 792 patients from 13 academic medical centers in the USA who differed in age, race, and baseline disability levels. However, there were no significant differences in sex, BMI, level of herniation, associated symptoms, smoking, hypertension, or diabetes. Considerable differences were observed in the reoperation rates, ranging from 4-21% at year 4, with recurrent disc herniation being the most prevalent cause of reoperation (2.7%). At four years, no differences were observed in body pain or physical function. Matsumoto et al. (2013) [35] used the JOA score to review patients who underwent microendoscopic discectomy (MED), a minimally invasive surgery, for lumbar disc herniation (LDH). LDH recurrence was 10.8% and more frequent among patients with caudal (19.0%) and rostral migration than those without migration (10.2%). Wang et al. (2012) [34] also investigated the clinical effects of MED for LDH therapy over the long term. They rated 5% of patients as having poor outcomes using the modified MacNab criteria. Only 3.5% of patients required reoperation owing to herniation recurrence. Mochida's approach resulted in a disc-height ratio of 76.25% on average. Around 67% of the patients were estimated to continue working in their previous capacities. They concluded that MED's long-term clinical results are superior to conventional discectomy. Majed Ali in 2021 [40] using North et al. 1991 definition [42] discovered that 11% of patients developed FBSS, which affected the daily activities of 54.8% of patients. Most patients with FBSS were in their 40s-50s (38.7%), followed by those over 60 (25.8%). The female-to-male ratio was 1.0:1.6. Regarding the secondary management for patients with FBSS, 61.3% underwent surgical intervention, whereas 38.7% received conservative treatment. Lee et al. (2019), using the FABER test, thigh thrust, iliac distraction test, Gaenslen’s test, and pain relief of >70% achieved from Sacroiliac joint (SIJ) block performed under computed tomography guidance, discovered that 12% of lumbar fusion surgery patients developed SIJ pain, with no difference between those who underwent one level fused versus those who had four or more levels fused [29]. Parker et al. (2010) discovered that 32% of 111 patients with single-level lumbar discectomy experienced mechanical back pain associated with same-level degeneration, which was linked to significant increases in healthcare expenses [28]. Weir (2017) [30] retrospectively studied a large population-based cohort of 10,216 patients from the CPRD and HES databases in England and reported that 20.8% of the patients had persistent post-surgical pain (PPP), which was associated with younger female patients with more comorbidities. PPP was associated with significantly increased rates of healthcare usage and pain medication costs. Bakhsh A (2010) [37] retrospectively reviewed 68 patients' medical records who had lumbar disc operation, of which 15% were deemed unsatisfactory. In 8.8% of patients, new neurological deficits manifested postoperatively as foot drop and calf muscle weakness [37]. Schoell et al. (2019) [26] surveyed a large nationwide insurance database of 70,581 patients and reported an FBSS prevalence of 15.05%, which was associated with increased age, depressive symptoms, multilevel procedures, and posterior fusion. Using the EQ-5D scale for measuring health-related QOL [43] and K6 [41], Inoue S (2017) [36] discovered that 20.6% of patients had FBSS, which was associated with multiple lower back and spinal fusion surgeries. FBSS was more prevalent in patients aged > 65 years and showed no sex differences. Respondents with FBSS had a lower QOL, lower satisfaction, and higher psychological distress or depression. The meta-analysis of a binary categorical outcome variable, “prevalence of persistent pain,” was conducted to assess the combined prevalence extracted from 16 published studies. The total sample sizes of these studies were 85,643. The pooled prevalence of persistent pain using the random-effect model was 14.97% (95% confidence interval: 12.38-17.76). The Cochran’s Q value (Q=490.57, dff=15, P<.0001) and I2 value (96.94%) were significant, indicating highly significant heterogeneity among the 16 studies. Hence, the pooled prevalence using the random-effect model was considered. Publication bias assessed using the Egger’s test (P=.9733) had no significance, indicating no publication bias (Table 2 and Figure 2).

**Table 2:** Meta-analysis for pooled prevalence of persistent pain after spinal surgery across the published studies

Author name and year	Sample size	Prevalence (%)	95% CI	Weight (%)	
				Fixed	Random
Bugis SM et al. 2022 [9]	231	21.645	16.513 to 27.520	0.27	6.60
Majed Ali et al. 2021 [40]	283	10.954	7.565 to 15.187	0.33	6.84
Schoell K et al. 2019 [26]	70581	15.049	14.786 to 15.315	82.40	8.16
Lee YC et al. 2019 [29]	317	11.987	8.624 to 16.081	0.37	6.96
Weir S et al. 2017 [30]	10216	20.801	20.017 to 21.601	11.93	8.12
Inoue S et al. 2017 [36]	1842	20.630	18.803 to 22.551	2.15	7.93
Matsumoto M et al. 2013 [35]	344	10.756	7.687 to 14.520	0.40	7.04
Desai A et al. 2012 [27]	792	2.652	1.649 to 4.025	0.93	7.63
Ahsan K et al. 2012 [38]	398	15.075	11.705 to 18.975	0.47	7.17
Marquardt G et al. 2012 [31]	87	27.586	18.541 to 38.212	0.10	5.02
Lubbers T et al. 2012 [32]	22	9.091	1.121 to 29.161	0.027	2.40
Wang M et al. 2012 [34]	151	5.298	2.315 to 10.172	0.18	5.99
Chumnanvej S et al. 2011 [39]	60	8.333	2.761 to 18.386	0.071	4.29
Parker SL et al. 2010 [28]	111	32.432	23.854 to 41.975	0.13	5.47
Silverplats K et al. 2010 [33]	140	22.857	16.190 to 30.708	0.16	5.87
Bakhsh A 2010 [37]	68	14.706	7.284 to 25.386	0.081	4.54
Total (fixed effects)	85643	15.646	15.403 to 15.891	100.00	100.00
Total (random effects)	85643	14.971	12.383 to 17.758	100.00	100.00

Discussion

This systematic review examined clinically relevant back and leg pains following spinal surgery. Most studies have shown that standard discectomy has long-term benefits despite residual LBP and recurrent herniation. Nevertheless, FBSS accounts for 5-27.6% of spine surgery despite advances in surgical technique [6,7]. Patients with FBSS experience worse pain, QOL, and physical function than those with osteoarthritis, rheumatoid arthritis, complex regional pain syndrome, or fibromyalgia [44]. Ikeda et al. discovered that 70% of Japanese individuals over 65 were impaired [45]. Pain intensity, physical function (QOL score), and disability are strongly correlated [44,1,45,46]. Patients may experience a peak in recovery for physical function and pain six months after surgery [47]. Most patients are satisfied with multimodal treatment strategies and multidisciplinary teams for pain management [48]. Improvements in patient outcomes for FBSS can only be achieved via the collaborative efforts of doctors, psychologists, physiotherapists, and other allied health practitioners. Experienced surgeons should select appropriate patients, diagnose, and address problems in the operating room [48,49]. Misdiagnosing the wrong level may lead to FBSS [40]; however, other factors can cause it. For example, poor psychosocial well-being is strongly associated with outcomes of lower back surgery [50]. Studies have also linked FBSS to lower socioeconomic and educational statuses due to their heavy workloads [45].The SPORT trial data from multiple academic institutions revealed that first-time discectomy had comparable nerve root damage, surgical mortality, SF-36, pain and physical function scores, and ODI after four years [27]. The sex disparity in FBSS may be attributed to degenerative spinal diseases, which are more prevalent among women [44,45,51]. Interestingly, sex disparities were observed in the outpatient management of post-laminectomy syndrome, as opioids were prescribed more commonly to men, whereas neuropathic medications and NSAIDs were used more frequently by female patients [52]. However, Majed Ali discovered that FBSS was low (11%), more prevalent in men, and the mean age was 50.3, while the most prevalent age range was 40-49. They attributed their findings to a higher youth population than other studies' populations [40]. This is contrary to other studies that reported greater impairment among patients of advanced age [45,53,54] and women [55]. Patients with obesity had a lower QOL than slim and active patients [56,57]. Obesity aggravates the pain and increases the risk of infection and hemorrhage, which can complicate surgery and precipitate FBSS [58,59]. Age over 50 years, particularly over 70 years, is a common risk factor for FBSS, associated with substantial disability and pain [45]. Smoking increases the risk of FBSS [58]. Follow-up care may impact FBSS; hence, patient management should involve referral and follow-up [30]. Reoperation is one of several options, along with other therapies, including exercise, physiotherapy, behavioral rehabilitation, medicine, interventional procedures, neuromodulation, and implanted technology. Avoiding inefficient and dangerous drugs in patients with difficult FBSS may improve outcomes and save treatment costs [60]. Residual symptoms can influence surgical satisfaction, QOL, and mental health. Preoperative communication between patients and doctors may help reduce the incidence of postoperative FBSS. Internet surveys are inexpensive, anonymous, and simple, thus reducing surgeons’ prejudice, although medical data may be unreliable. However, online resources for FBSS are deficient in quality and quantity [61]. Furthermore, patient-reported outcomes can enhance QOL and satisfaction in many healthcare institutions. Therefore, anonymous third-party internet surveys may be used to evaluate patient treatment satisfaction [29,36]. Large data analysis from hospitals and primary care providers demonstrated that one in five patients who underwent lumbar spine surgery in the United Kingdom developed PPP, which increased resource consumption and expenses. Unlike studies that used unrepresentative patient samples, this study may have improved the prediction of lumbar surgery PPP [30]. MED minimally invasive surgery yields a long-term recurrence and reoperation rate of 10.8%, equivalent to conventional discectomy [35]. Surgeons should address variables that may increase FBSS rates and evaluate treatment options, such as poor mental health or preexisting depression, which are predictors of LBP [62]. Workers' compensation patients had the poorest spinal surgery outcomes. Smoking and obesity worsen spinal surgery outcomes [63,58]. Also, inappropriate spinal surgery choices, such as wrong-level decompression, may have led to poor outcomes in some patients. Postoperatively, an early recovery may be hampered by complications such as epidural or subdural hematomas, infection, pseudomeningocele, or nerve injury. In the long term, changed biomechanics in the spine can lead to load distribution changes and accelerate the deterioration of spinal segments nearby the lumbar fusion, causing additional pain. This study has some limitations. The literature on LBP prevalence and other complications following lumbar discectomy is limited, with significant heterogeneity. We narrowed our search to 2010. Most studies were conducted retrospectively with small, non-representative samples. The outcomes were not standardized among the studies. Using various measures, the researchers indicated the frequency of persistent back and leg pains among study participants.

## Conclusions

Despite technological and surgical advancements, FBSS affects many patients, accounting for 5-27.6% and a pooled prevalence of 14.97%. FBSS is a complex illness with multiple known and unknown etiologies. It is associated with increased age, female sex, depressive symptoms, multilevel procedures, and posterior fusion. Respondents with FBSS experienced a lower QOL, lower satisfaction, and higher psychological distress or depression. PPP significantly increased healthcare and pain medication costs. No differences were observed in body pain or physical function during the long-term postoperative period, regardless of the surgical center. Appropriate preoperative communication, as well as multidisciplinary and coordinated treatment strategies, yielded the best results. FBSS impairment necessitates proper patient selection. In addition, surgeons must consider poor prognostic indicators, including psychological factors. Residual symptoms can influence surgical satisfaction, QOL, and mental health. Preoperative communication between patients and doctors may help reduce the incidence of postoperative FBSS. MED was associated with LDH recurrence and reoperation rates equal to those of conventional discectomy. Prospective longitudinal studies examining the incidence of recurring degenerative back pain, disability, and QOL using standardized methodologies and validating outcome instruments are required.
